# Online polarization and cross-fertilization in multi-cleavage societies: the case of Spain

**DOI:** 10.1007/s13278-022-00909-5

**Published:** 2022-07-13

**Authors:** Rubén Rodríguez Casañ, Enrique García-Vidal, Didier Grimaldi, Carlos Carrasco-Farré, Francisco Vaquer-Estalrich, Joan Vila-Francés

**Affiliations:** 1 Internet Interdisciplinary Institute, Universitat Oberta de Catalunya, 08018 Barcelona, Catalonia Spain; 2 IDAL, Intelligent Data Analysis Laboratory, Electronic Engineering Department, ETSE, University of Valencia, 46100 Valencia, Spain; 3 Department of Management, University Ramon Llull –la Salle Business School, 08022 Barcelona, Spain; 4 Information Management Department, Toulouse Business School, 08019 Barcelona, Spain

**Keywords:** Echo-chamber, Group polarization, Social media, Multi-cleavage, BERT, Artificial intelligence

## Abstract

The impact of the social media (SM) has been seen on the one hand as the cause of large exacerbation of negative messages, responsible for massively harmful societal phenomenon against democracies. On the other hand, recent studies have begun to look at how these online channels were able to provide a new impulse in human communication. The novelty of our work resides on analysing several axes of polarizations related to different societal topics. We believe our approach to reflect a more complex society, differing from the recent literature, which has considered a unique left–right dichotomic cleavage. Our methodology consists of extracting topics from the priority themes of the SM debate, using BERT language processing techniques and TF-IDF model. Our results show situation of social media interactions in a multidimensional space does exist. We highlight how social media behaviours, polarization and cross-fertilization differ as upon concrete topics. We argue therefore the ‘mega-identity partisanship’ which differentiate US online users in two different spaces cannot be extended for the rest of countries taking as first evidence the case of Spain. Further research should extend our conclusions for a possible generalization.

## Introduction

January 6, 2021. A mob of protesters gathers around the US Capitol protesting for what they considered a stolen election. Through social media platforms like Twitter and Facebook, President Donald Trump called to action: ‘If you don't fight like hell, you're not going to have a country anymore’ (Woodward 2021). The situation escalates and the riots start, with hundreds of protesters assaulting the Congress, causing the death of a police officer and injuries to 138 more. Soon after, Donald Trump was permanently banned from Twitter. However, this was not the only case where online polarization—the extent to which people dislike their political opponents (Iyengar et al. 2012)—translates into offline consequences.

Other societal issues (e.g. Brexit, COVID vaccine) have generated large divisions of people opinions. This divergence in opinions observed in social media is known as ‘echo-chambers’ i.e. patterns of information sharing based on a set of strong political beliefs which impedes the introduction to opposing political views (Becker et al. 2019). While extant research exists about the role of social media echo-chambers on social polarization, there is a lack of agreement between empirical results (Iandoli et al. 2021). Some authors point them as the cause of large exacerbation of negative messages, while others differ. For example, Becker et al. (2019) argue that social media are responsible for massively harmful societal phenomenon against democracies (such as the Capitol assault). On the other hand, there is also evidence arguing the echo-chamber impact in some topics is overstated and that the degree of ideological segregation in social media may be overestimated because social media platforms allow people to have access to people that otherwise they would never connect to or be able to see their opinion (Barberá et al. 2015). Moreover, critiques to the importance of echo-chambers add that people have largely access to enough information via different media sources to fact-check and reduce the possible negative effect of echo-chambers.

Considering the opposed evidence, we shed light into the empirical paradox by proposing a more complex analysis that overcomes some weaknesses in previous literature.

One of the main feebleness of previous literature is that most of the papers have analysed the social media messages across one axis of separation (e.g. Liberals versus Conservatives). This is known as a cleavage: a “compounded divide” based on interests, opinions or attitudes that gives collective identity to its members and a strong organizational base (Bartolini 2005; Deegan-Krause 2006, 2007). Examples of cleavages include the separation between religion and the State, rural vs urban politics, center vs periphery issues, integration vs multiculturalism, or right- vs left-wing politics among others (Lipset and Rokkan 1967).

However, the novelty of our work resides on considering several axes of polarizations related to different societal cleavages (also referred as topics), reflecting a more complex and multi-atomised society. Besides the most usual one (left-versus right-wing parties), our paper studies polarization and sentiment towards other social and political communities, potentially revealing a mixed result between both research evidence: polarization may arise in some topics while cross-fertilization-interchange of ideas and points of view through information diffusion patters (Bakshy et al. 2015)- may appear in others.

Among others, we focus on polarizing topics like social postulates (LGTBI rights, environmental issues, social rights) or territorial organization positions (Catalonia independence vs Spain centralization). Consequently, our research question consists of investigating whether social media platforms like Twitter act as either an echo-chamber characterized by selective exposure, ideological segregation and political polarization, or a widespread and national conversation in which citizens of different ideological perspectives read and retweet one another’s messages in a cross-fertilization mode.

To disentangle the dichotomy of previous literature regarding the role of social media echo-chambers in group polarization, we operationalize our analysis through three Research Objectives:

1. Revealing a multi-cleavage context by identifying topics discussed in social media during a polarized presidential campaign.
2. Identifying the polarity of sentiments in online out-group and in-group communication.
3. Understanding the topics of cross-fertilization of social media echo-chambers.

As per our empirical setting, we select the special case of 2021 Catalan Presidential elections due to the high-polarized environment situation that this election provided us (Grimaldi et al. 2020).

## Literature review

### Social media context in Spain

Catalonia is a region in north-east Spain with about 7.5 million people and its own language, parliament, flag, and national identity. It has also its own police force and controls some of its public services. Its capital is the city of Barcelona. Catalan nationalists have long complained that their region is treated unfairly by Spain, as most taxes are collected by Madrid and many of the social, cultural, environmental, and economic laws approved by the Catalan parliament have been withdrawn by Spanish courts.

In a referendum on 1 October 2017, declared illegal by Spain's Constitutional Court, about 90% of Catalan voters (turnout 43%) backed independence of Catalonia from Spain. The social environment of Catalonia since that referendum day has become more and more ‘explosive’ and highly polarized: because (a) there were important clashes when Spanish national police tried to prevent people voting and consequently a lot of people were injured; (b) after a high-profile trial in 2019, nine Catalan political and social leaders were all handed prison sentences up to 13 years for the offence of sedition, including the Speaker of the Catalan Parliament and two social activists.

This failed bid to create a new state in the heart of Europe caused the worst political crisis in Spain since the failed military coup in 1981. In 2021, the partial pardons offered by the president Sanchez for the nine politicians instead of cooling off the atmosphere have also reactivated the debate and the polarization of the Catalan versus Spanish feelings (Derqui and Grimaldi 2020). Some 63% of the Spaniards said they were against granting these pardons, while 25% were in favour (Tomàs 2021).

Most of the opponents are the conservative party called “Partido Popular” (PP), the far-right “Vox” and the right-wing “Ciudadanos” (Cs) party. In favour of the pardons, there are all the parties of left wing: “PSOE” (the historic socialist party led by the Spanish President Sanchez), “Podemos” (UP) the far-left party (as upon the national Spanish institute of statistics defines it) and the 4 nationalist Catalan parties independently of their economical line i.e. “ERC” (left nationalist wing), “CUP” (far-left nationalist wing) but also “PDeCAT” the Catalan European Democratic Party (right nationalist wing) and “JxCat” (Junts per Cat, the liberal nationalist party). This situation offers us a strong case study of a highly polarized social climate and puts in evidence the interest to search for complex echo-chambers structured by socio, economic, health and nationalist positions and topics.

### Social media generates multiple echo-chambers

In Social Science, group polarization (GP) describes the situation where members of a defined group end up a discussion by being more extreme in their position about the given issue (Moscovici et al. 1972). GP has been largely studied in offline settings since the 1960s (Myers and Lamm 1976). In the last decade or so, studies have begun to look at how Social Media (SM) were able to provide a new impulse in human communication (Matuszewski and Szabó 2019) and the potential impact on the configuration of the social and political debate (Barberá et al. 2015; Barberá and Rivero 2015; Iandoli et al. 2021).

Many European and American societies have seen how societal issues such as LGTBI (Lesbian, Gay, Bisexual, Transgender, Intersexual), vaccines (Giese et al. 2020), immigration, Islamism, social protection costs have generated large divisions of people opinions (Guijarro-Ojeda and Ruiz-Cecilia 2019). The experiment of Bail et al. (2018) shows how SM exacerbates this GP by creating ‘echo-chambers’. The analysis of the representativeness of SM users shows that societies can no longer be considered as the configuration of the Chamber of Deputy look like “physically” with its clear division between Left- and Right-wing parties or Liberals and Conservatives. Barberá et al., (2015) and Barberá and Rivero (2015) corroborate this point commenting that SM users can be indeed divided into different lines of demarcation such as geographical (between rural and urban zones), gender, multiplying the number of potential echo-chambers in the online version of these societies.

Our research extends their current studies analysing the interactions between the different echo-chambers while analysing the engagement on social media during the last Catalan presidential campaign. It participates into this debate, looking for different lines of demarcation which divide the social media. The first goal is therefore to determine the characteristics or topics of a multi-cleavage and complex societal landscape.

### Social media could provide positive and negative impacts

However, some scholarship studies (Messing and Westwood 2014; Prior 2013) suggest that social media allow users to be exposed to a variety of messages, larger than those exchanged in their “real” and physical life. They add they can be in contact with ideologies which their personal network has weak ties. As an interesting result, Bond and Messing (2015) argue it could consequently allow estimating ideology of users based on their preferences and attitudes in the SM (like or do not like; endorse or do not endorse a post). However, the debate about the relationship between GP and the exposure of political diversity is still progressing. Some authors estimate the cause-and-consequence effect could be to moderate and reduce it (Barberá 2014; Iandoli et al. 2021), while others believe the opposite (Bail et al. 2018; Prior 2013) and claim for a more aggravation of the political atmosphere.

In a bi-modal society configuration hypothesis, a sentiment analysis led by Rathje et al. (2021) shows a direct contact with animosity topics in an echo-chamber drives an “angry” reaction from other communities (out-groups) and leads to huge reaction on social media. This effect reveals to be the strongest amongst other emotions conveyed by topics (Druckman et al. 2021). These studies show also there is usually a positive bias to share positive content inside its proper communities (Milkman and Berger 2014). It seems indeed SM reflects the desire to maintain a positive self-presentation to an individual’s own community (Van Bavel et al. 2021). However, a gap still exists and to our best knowledge, few studies have realized an exercise of sentiment analysis considering not only a simple one-axis model but preferably a multi-cleavage society landscape. Our paper fills this gap not only by analysing the polarity of the sentiment, but also the topics which foster online out-group and in-group engagement.

### Permeability of the echo-chamber

However, recent works have recently participated in the topical debate about the non-permeability of SM community groups and the existence of SM clusters highly bordered by partisan lines. Barberá et al. (2015) argue that the bad aspect of the echo-chamber concepts is exaggerated. Indeed, they argue citizens who are interested in a societal topic, largely fear partisan monopolistic view which is present in an echo-chamber. Consequently, these latter tend to avoid it. The authors add that someone confronted to an inconsistent or insufficient news can have access to other platforms or media to contrast this information and fact-check it, by employing a search engine in his/her mobile or personal computer. This use of multimedia platforms tends to reduce the isolating effect of echo-chambers (Ahlers 2006; Dutton et al. 2017). However, literature is scanty to investigate the propensity of the cross-ideological dissemination, i.e. if an SM user highly interested in a societal topic tends to stay in one echo-chamber. Dubois and Blank (2018) cover it, but considering the societal interests without splitting them into the different political topics conveyed in SM. Our paper extends this analysis, and our third research objective consists of looking for the themes which may decrease or increase the permeability of echo-chambers in a multidimensional society environment. Our objective is to evaluate how the echo-chambers in multi-cleavage societies exist, in concordance with one literature stream, but are also overlapping (not isolated) as it is stated in the other literature stream.

In a nutshell, our paper raises the question of how social media polarizes the societies and how the phenomenon of cross-fertilization of echo-chambers works in complex multi-cleavage environments.

## Methodology

The methodology applied to answer the research question and obtain the objectives, as set before, are detailed below. The procedure followed is described in the following flowchart. Each of the phases is explained in each subsection of the methodology chapter.
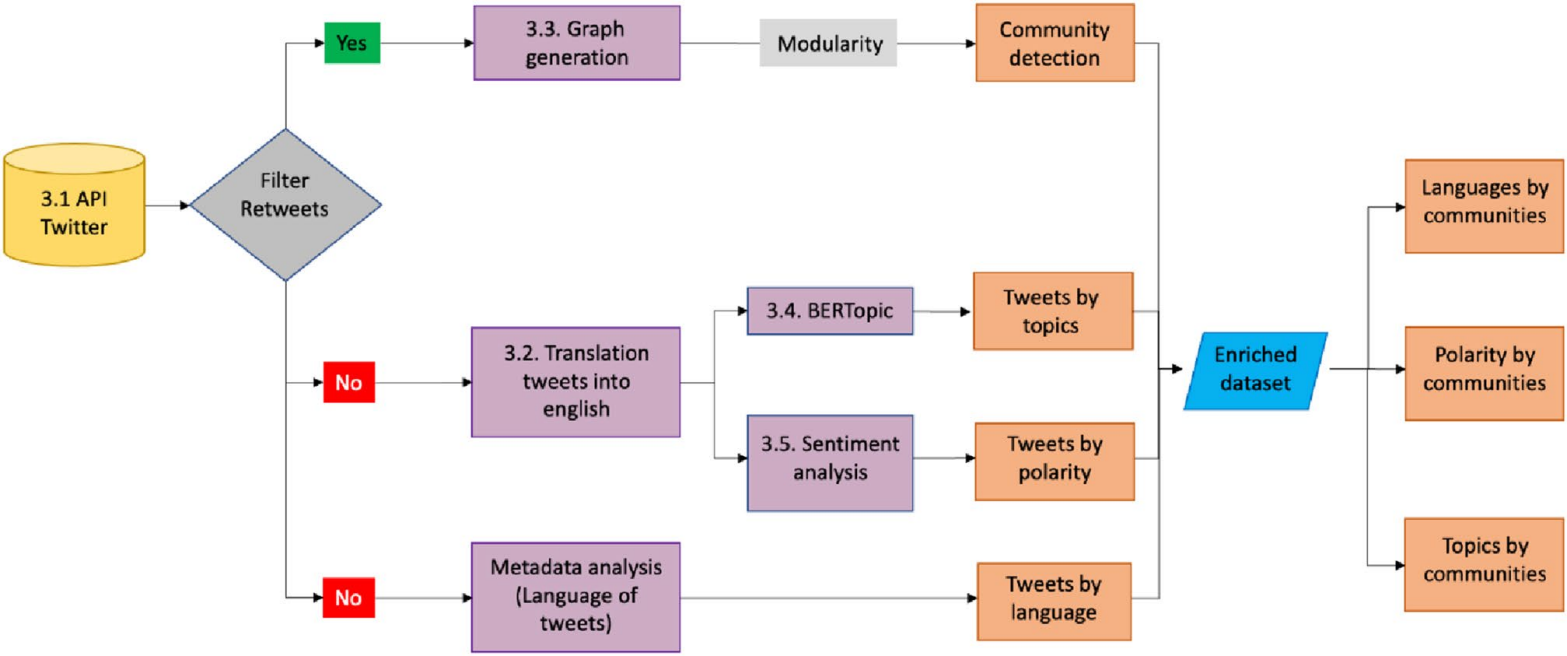


The process followed in this research work was composed of different subprocesses. Once the set of tweets was collected, in parallel, we worked in independent subprocesses whose results were unified for later analysis. First, a graph was made with the retweets and by applying modularity multiple times, we defined the community associated with each user and therefore to each tweet. On the other hand, the tweets were translated into English to normalize the language and thus be able to apply two NLP models, bertopic to associate a topic to each tweet and polarity sentiment to associate a degree of polarity to each tweet. Finally, an analysis of the metadata provided with the Twitter API was made in order to join all the relevant information with the results of the other processes so that we could finally answer the questions we have raised in the introduction.

### Dataset

The collect of data was done by using hashtags, ensuring the context of all the tweets gathered is related to the campaign. The list of hashtags is in Appendix 1. First, the extraction and provenance of the data is discussed. The initial data set is made up of 501,027 tweets identifiers generated during the 2021 elections to the Parliament of Catalonia, between the dates of January 24 to February 14, election day. During that period, the different hashtags used by each of the political organizations that were presented for the vote have been monitored.

With the identifiers of the tweet, the academic Twitter API is used with the goal of obtaining fields such as the text, the publication date, the account that writes each tweet or the type of interaction (retweet, quote, reply or original tweet). This information will be very useful in the next steps to generate relationships between users.

### Data cleaning and preparation

After extracting the whole information for each tweet, a standardization of the language that allows us to start the analysis from the same starting point is needed. The initial dataset includes tweets in Spanish, Catalan, and English. For this purpose, Google's API Translation allows the language of each of the texts to be automatically detected and translated into English. The common language of the normalization chosen is English because the current natural language processing models, as well as the models for cleaning the data used, come up with better performance in this language.

After normalizing the data, a graph was generated to identify the different communities that served us to measure the interaction between each other. Later, sentiment analysis was carried out to determine the level of polarization in the messages of each community and, finally, the implementation of a topic modelling model with which to determine the most discussed topics.

### Network creation and community detection

To generate the graph, the first action is, taking the interactions through retweets between individuals (i.e. Retweeted Network), to create an adjacency matrix between each of the users with respect to the rest of the individuals (Finn et al. 2014). In a way that, the people who interact the most with each other can be measured by the number of retweets that are made. Once the matrix has been generated, the Bastian et al. (2009) software is used to create the interaction graph between accounts, using a Force Atlas 2 distribution. After generating the graph, the next step is to use clustering methods (modularity) that allows us to differentiate communities.

Modularity is the fraction of edges that fall within the given groups minus the fraction expected if the edges were randomly distributed. It has identified nodes to communities using the default values of the modularity algorithm and identifying communities through the main accounts of each party (*'@populares', '@socialistes_cat','@CiutadansCs','@EnComu_Podem','@vox_es','@cupnacional','@RecortesCero','@Esquerra_ERC','@JuntsXCat','@Pdemocratacat'*). These accounts are located near the cluster centroids and are among the most prominent accounts within their cluster along with the candidates' accounts. Modularity has difficulties in assigning a community at boundary nodes with few links. Knowing this and to reduce this possible error, it ran 5 modularity simulations and only associated nodes to a cluster if in 4 of the 5 simulations they had chosen the same community. Doubtful nodes (not fulfilling this condition) were ignored from the subsequent analysis since they were not associated to any community, although they were determinant in the composition of the retweet graph.

After laying the foundations through the graph, we proceed to analyse two interesting points that will allow us to propose new lines of analysis taking into account different cleavages (topics). At this point, we start from two data sources: on the one hand, the result of the clustering obtained from the graph and, on the other hand, the identification of cleavages and the sentiment analysis (Grimaldi et al. 2019).

### Topic modelling with BERT

BERT is, nowadays, the most powerful model in language processing. It is based on transformers (Vaswani et al. 2017) and can solve general language problems such as translation, features and entities extraction, sentiment analysis, etc. (Devlin et al. 2019). Transformers have two blocks: encoding (which is responsible for correctly processing and encoding the input text) and decoding (which is responsible for encoding the output of the text).

In BERT, the input text is always coded with two elements depending on the problem to be solved (two sentences, a paragraph, and a question…). A start token is included at the beginning and, to separate the two elements, a separation token. Three encodings are generated for each word: an embedding, a position vector (indicates the relative position of each word within the phrase) and another embedding that indicates the segment to which it belongs (the first phrase includes an encoding and the second, another different). These representations are finally added to a new vector, processed by BERT. The training has two phases. In the first, the model learns to encode each word in a bidirectional way, considering what is around each word. This is accomplished by having the model fill in sentences with missing words. The next step is to train the model to learn to differentiate whether the continuation of the first sentence given is the second sentence given to it. Once these two sentences are completed, the model is ready to be able to solve any task, adding extra layers and fine-tuning them for each type of problem.

As said, this method allows us to extract the most relevant topics in the public debate, as well as their relationships and interactions between the different communities. For its implementation, transfer learning techniques have been used with the aim of reducing training times and obtaining more precise results. Bertopic combines BERT technology and the TF-IDF model (Grootendorst 2020). Summarizing, the model generates a numeric vector that encodes each text input (in this case, each tweet) based on its semantic characteristics, known as document embedding. From there, the topics groups are extracted and grouped into the most similar ones using clustering models. Finally, the result of the clustering is passed through a TF-IDF model from which the most relevant words of each topic are extracted.

### Sentiment analysis

The aim at this point is to analyse the polarity of each of the messages belonging to each topic for each of the communities. To do this, and after crossing data between clustering and tweets, a pre-trained model is used (applying, again, transfer learning techniques) called Hugging Face Bert Base Multilingual Uncased Sentiment (Hugging Face 2021). This model, based on the transformer architecture, generates a score as an output between one and five for each of the tweets that are passed to it, one being a message with the maximum negativity and, five, a positive message. In this way, it is possible to carry out an exhaustive analysis on the polarity of the discourse for each of the communities obtained in the community graph.

## Results

The results aim to answer how the phenomenon of cross-fertilization of echo-chambers works in complex multi-cleavage environments. To do so, we present our results following the Research Objectives described before: First, revealing a multi-cleavage context by identifying topics discussed in social media during a polarized presidential campaign. Secondly, identifying the sentiments that foster/suppress online out-group and in-group communication. Finally, understanding the mechanisms of cross-fertilization of social media echo-chambers.

### A multi-cleavage society landscape reflected in social media

The following analysis focuses on the interactions between the different political parties that stand in the elections, with the aim of identifying new axes that define the political changes that are taking place in our society.

**Fig. 1:**
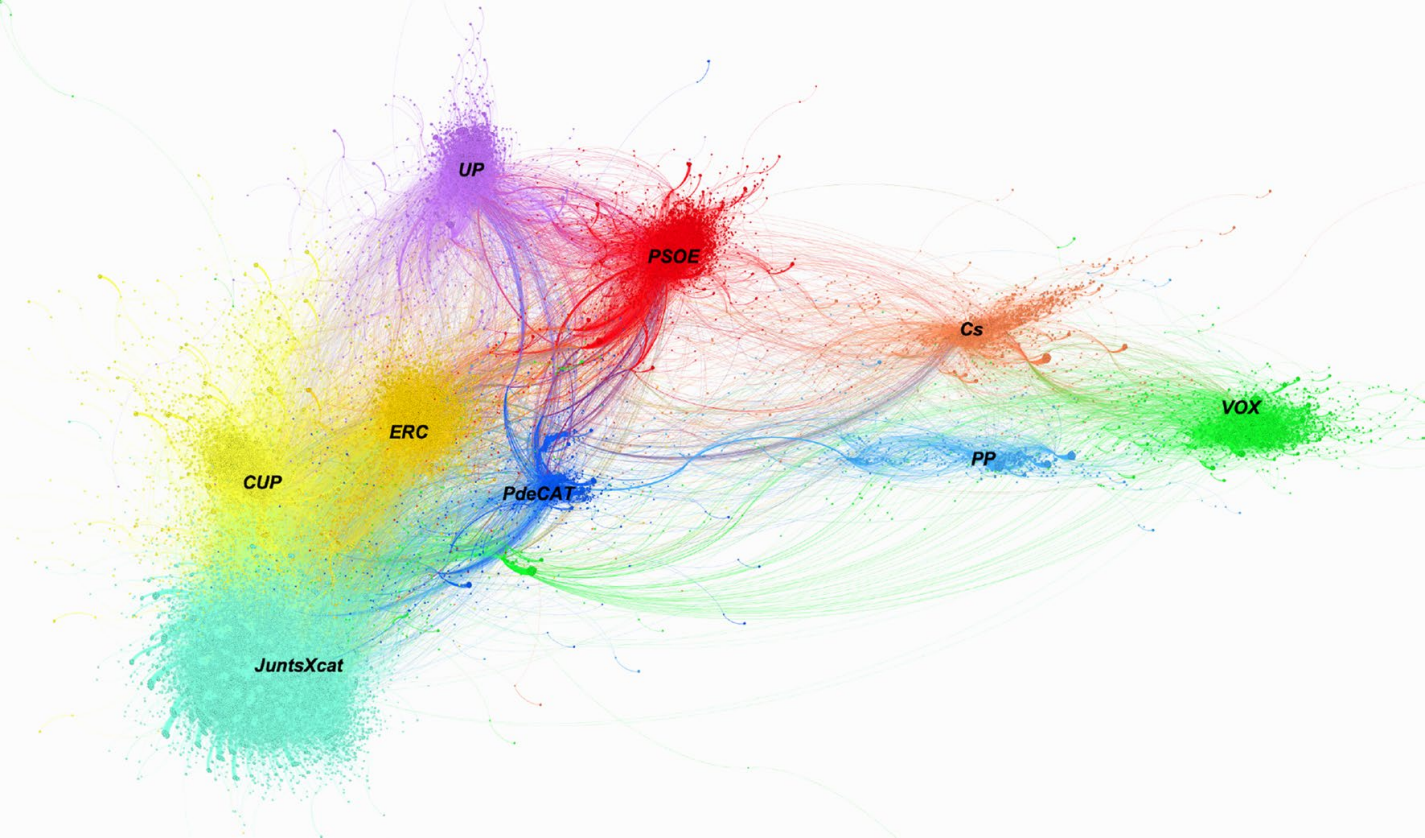
Graph of RTs on the Catalan campaign February 2021 coloured by modularity

Figure 1 generated by the retweets creates multiple communities where the party accounts are the accounts that have received the most retweets within their group. The proximity between them reveals that there are more users who act as a bridge between two communities. Starting from this idea, the graph shows the harmony between users for the Catalan elections context. Also, elections involved in the midst of the pandemic have drawn two axes.

The first is the classic left and right axis and, the second is the Spanish nationalism-Catalan nationalism axis. For this context, it is observed that the left, regardless of their territorial position, are closer to each other than the independence rights against Spanish nationalists. For this reason, the axis of greatest polarization for these Catalan elections has been the territorial question.

From a territorial perspective, the ‘*PSOE*’ and ‘*UP*’ more inclined to dialogue with a position of recognizing Catalonia as a territory with its own identity within Spain are in more neutral positions within the network. Right in the other region of the graph, there are the most nationalist Spanish parties, where the ‘*PP*’ and ‘*Cs*’ are closer to each other than to ‘*VOX*’, whose community is displaced to one end of the network.

The modularity algorithm identifies a communityfor each node, up to 5 calculation simulations were performed and only when 4 out of 5 occasions the algorithm provided the same community, then we consider that node belongs to the community defined by modularity. The average modularity obtained had a value of 0.73. Once our graph was divided by communities, we were able to identify each community by observing that the most prominent accounts are the accounts of the main parties as well as the accounts of political leaders. Once the political party was identified, each community was coloured with the reference colour of each party.

In order to contextualize the ideological position (left vs right and independence vs centralism) of the different parties that appear in the analysis, a descriptive table has been added to Appendix 2.

### The language as other “game changer”

**Fig. 2:**
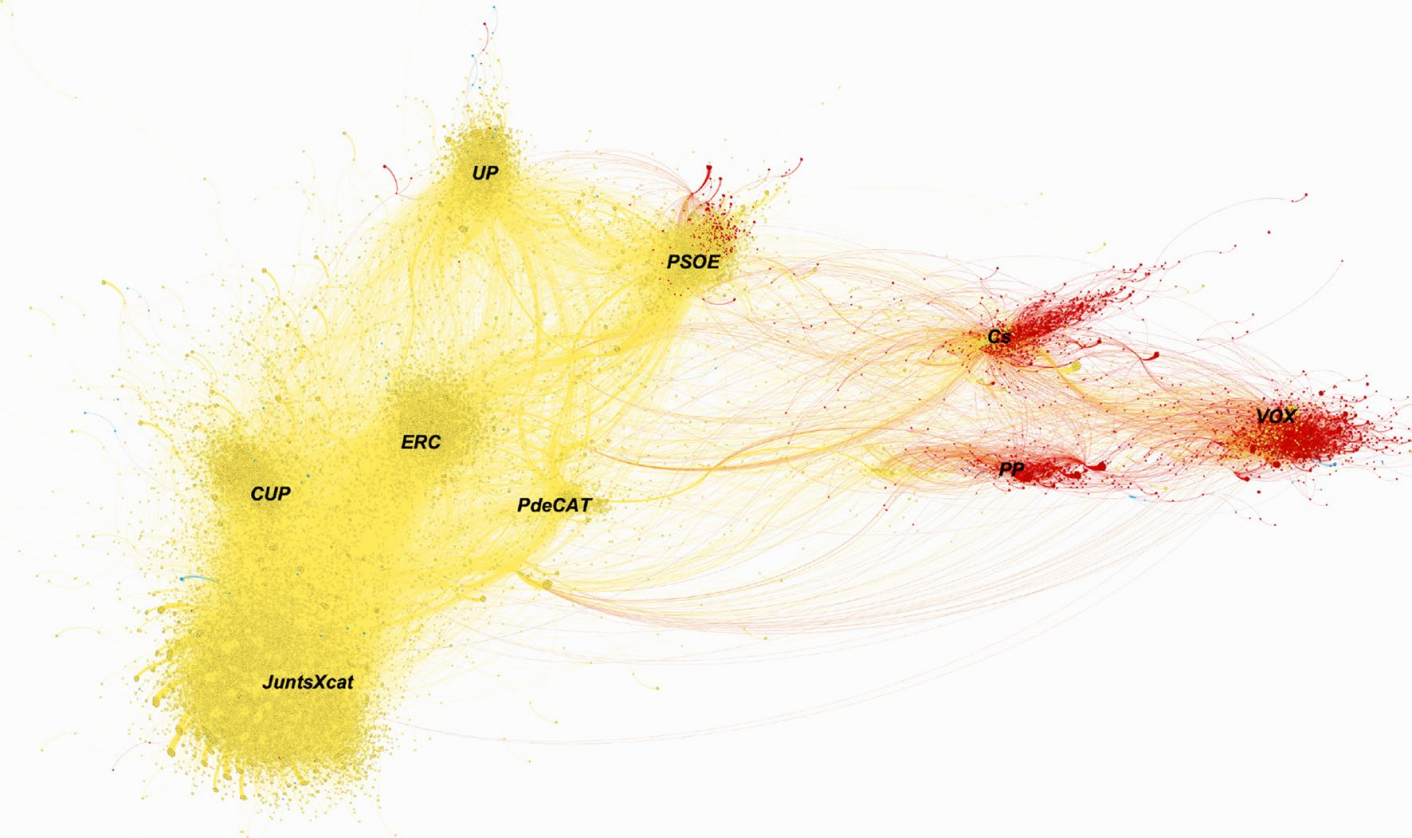
Graph of RTs on the Catalan campaign graph colored by the user's preferred language

In Fig. 2, the parties of the Spanish nationalist group, mainly conservative, post mainly in Spanish, giving greater weight to their national identity. On the other hand, the parties with a more progressive and independent identity give greater weight to the autonomous language, Catalan.

Emphasizing the idea that the territorial axis has been more decisive for the positions of the parties within the network / Twitter, we can plot each user by the preferred language (Red = Spanish, Yellow = Catalan and Blue = English). Thus, we analyse the graph from the point of view of language.

Visualizing the graph by the preferred language, two large blocks are observed. On the one hand, the most nationalist Spanish parties (‘*PP*’*, *‘*Cs*’* and *‘*VOX*’) and, on the other hand, the Catalan independence group (headed by ‘*JxCat*’, ‘*ERC*’ and ‘*CUP*’) and non-independent progressive parties, such as the ‘*PSOE*’ and ‘UP’. Furthermore, within each community, the users whose preferred language is Catalan are closer to the centroids of the pro-independence groups.

**Fig. 3:**
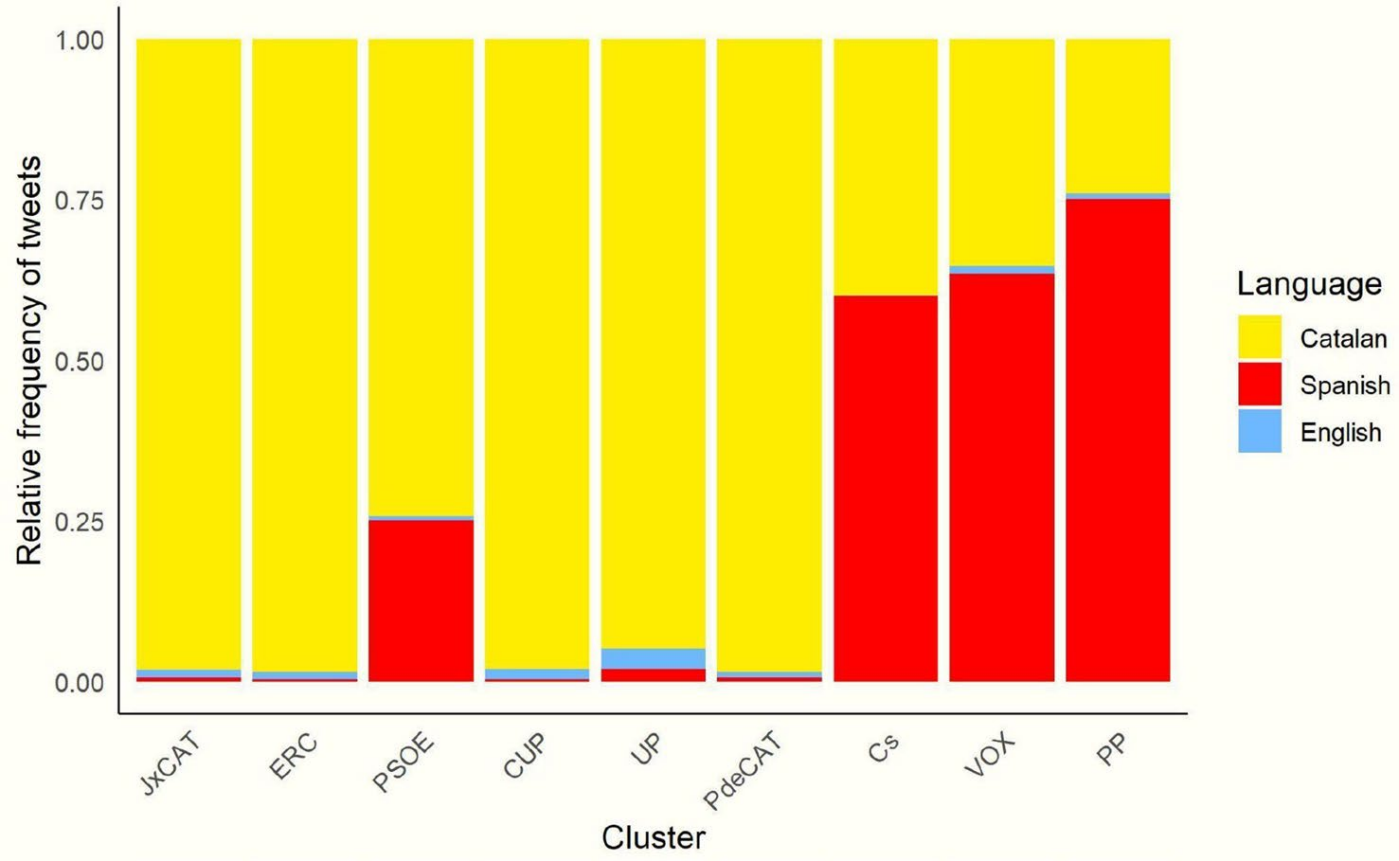
Presence of languages by community

To determine the presence of Catalan, Spanish and English in each community, we calculated the percentage of tweets written in each language by community (Fig. 3).

**Fig. 4:**
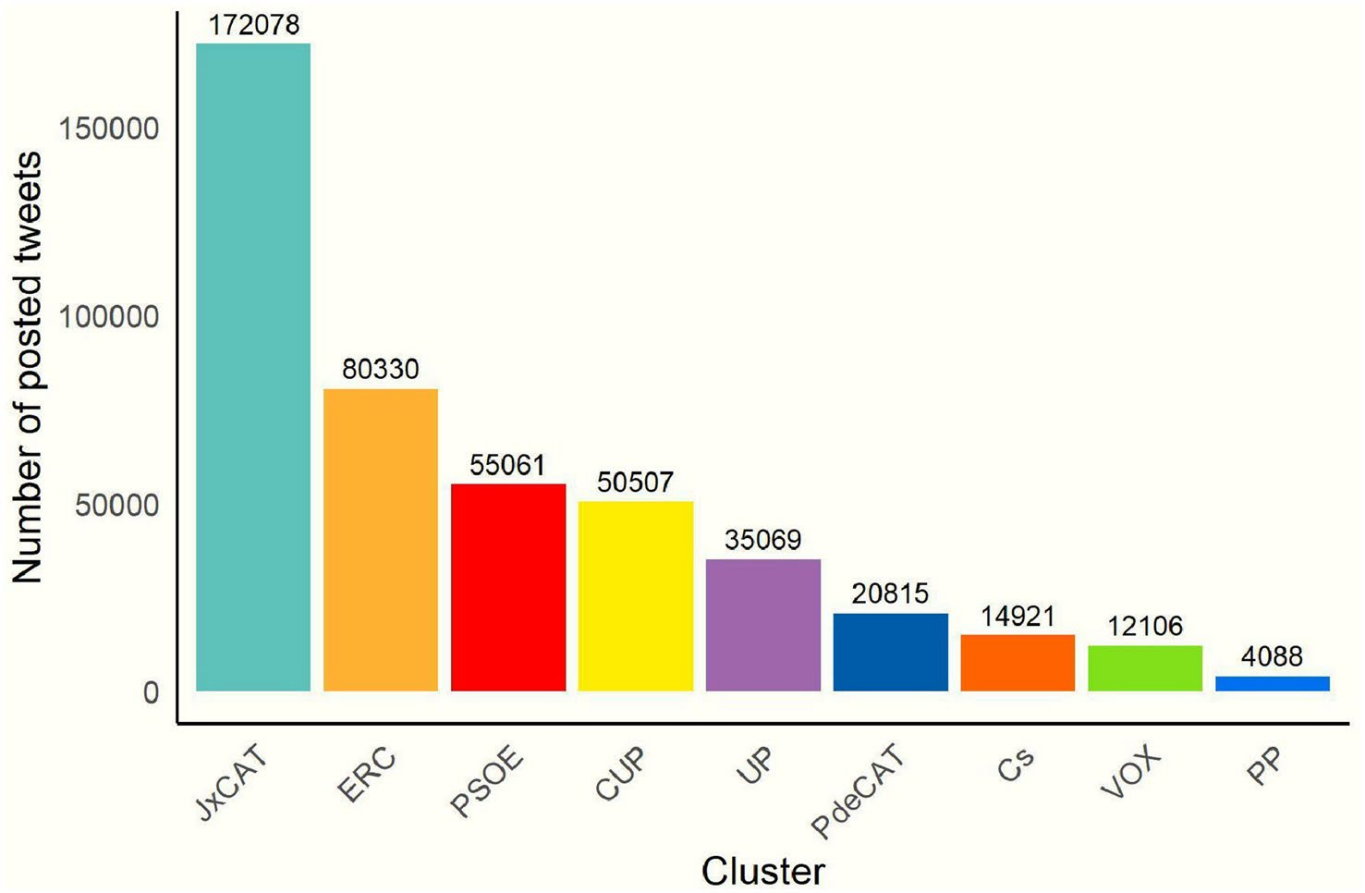
Number of generated tweets per community

By identifying the community to which each user belongs, the weights of each group—in terms of number tweets—can be seen in the election debate. By counting the tweets written by users from the different communities, we identify a high weight of the parties considered pro-independence (‘*JxCat*’, followed by ‘*ERC*’, the ‘*CUP*’ and ‘*PdeCAT*’) as shown in the Fig. 4. Next ones are the national progressive parties: ‘*PSOE*’ and ‘*UP*’ (with less weight).

It should also be noted the little presence of the more conservative parties (and more biased towards Spanish nationalism) in terms of communication level. The weight in the number of tweets from ‘*VOX*’, ‘*PP*’ and ‘*Ciudadanos* (*Cs*)’ is lower. This may be due to several reasons: the less relevance and political presence that these parties have in the Catalan territory, or that the less presence of these parties may be linked to the political strategy, giving greater weight to this confrontational discourse at the national debate and not so much at the regional debate. In other words, the discourse of the Catalan independence and nationalist axis, by the most conservative parties, is transmitted more easily to the rest of Spain.

### Sentiment analysis per community

Another aspect that has been conditioning national politics has been hate speech, focused on issues such as feminism, independence movement and the defence of language.

**Fig. 5:**
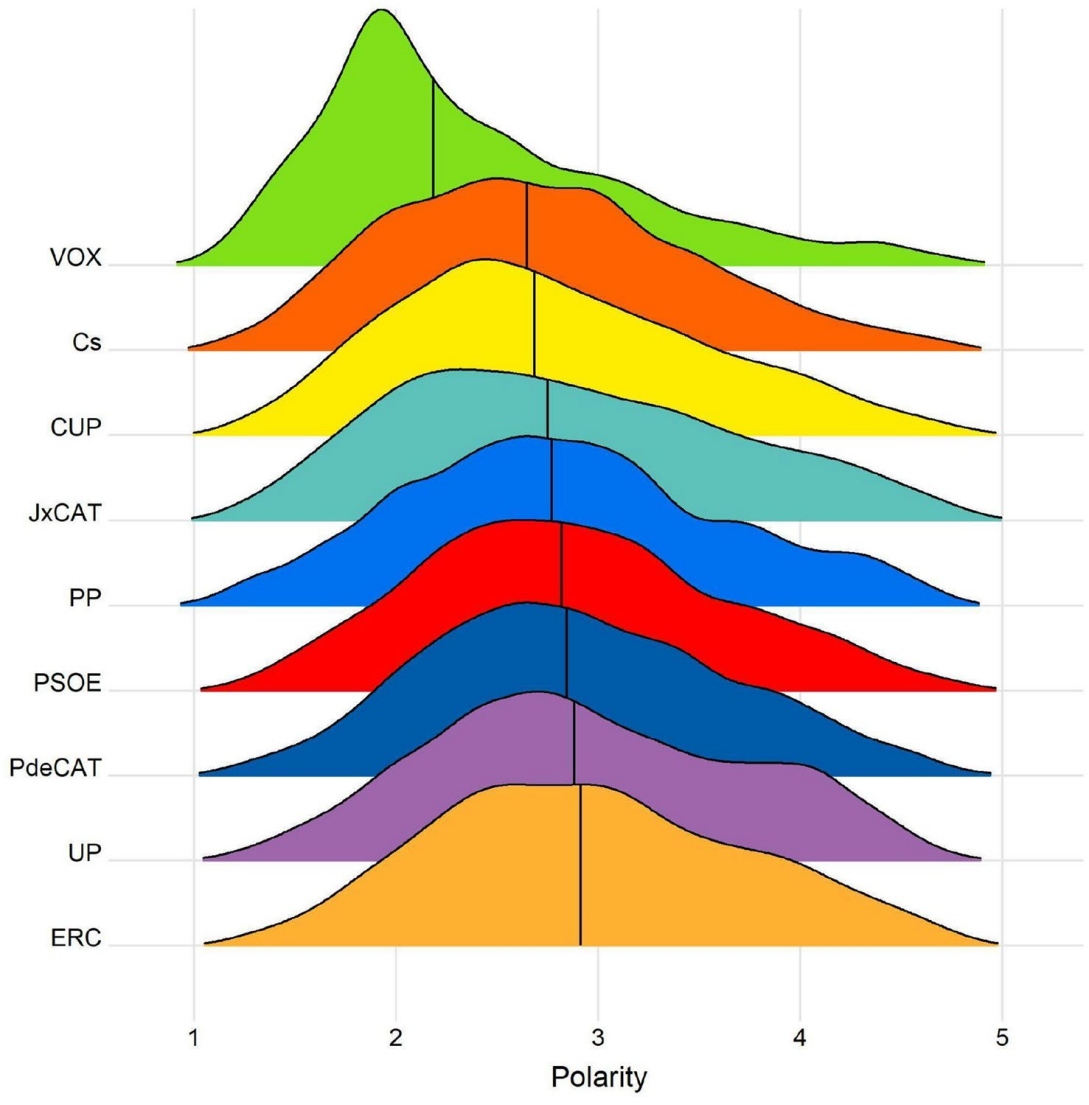
Polarity distribution in each party's tweets

Figure 5 shows a distribution of the polarity of the tweets belonging to each of the political groups, with one being the value assigned to the most negative messages and five being the value assigned to the most positive messages, as it has been exposed in the *Sentiment Analysis* paragraph.

As it can be seen in Fig. 5, there is a clear difference between ‘*VOX*’ and the rest of the parties. Most parties are centred between 2.5 and 3 and ‘*VOX*’ is more inclined to more negative messages. Although ‘*VOX*'s presence on Twitter during the Catalan campaign was less, their messages carried a much greater negative and confrontational component.

### The mechanisms of cross-fertilization in social media echo-chambers

The implementation of the Bertopic model on the set of tweets has allowed us to identify 45 campaign topics. As a whole, we had a table composed of the tweet, the user of the tweet and their membership in a community, the polarity of the tweet and now the most likely associated topic. With this, we can create aggregate tables, identifying the most common topics among different parties together with the most characteristic and differentiating topics of each community.

**Fig. 6:**
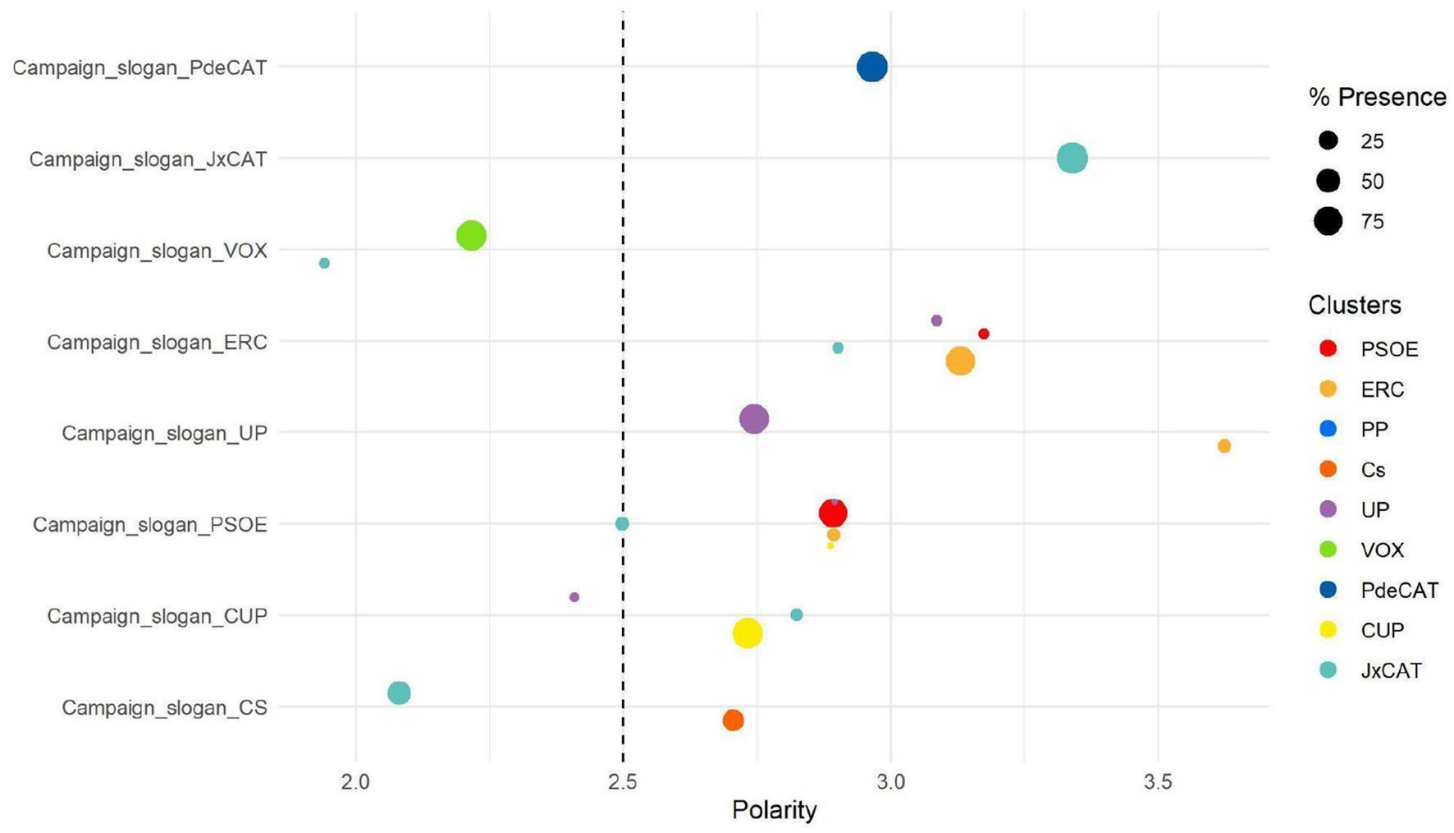
Presence of parties in campaign slogans

Thus, and as expected, if we select the topics whose presence of a party is greater than 80%, we find the vast majority with the campaign slogans. With campaign slogans, we refer to issues whose keywords are the name of the candidate, or the campaign hashtags of each party (Fig. 6).

In general, except for ‘*VOX*’, messages with campaign slogans in political groups have a greater presence and greater positivity. However, in ‘*VOX*’, some hashtags used had a negative charge on the adversary and therefore, their messages, even in the slogan ones, were negative. Among the most frequently topics used by the different communitiesare Covid-19, the debate between candidates, surveys, and trends. These issues are the ones that have a more equal presence among most of the parties.

**Fig. 7:**
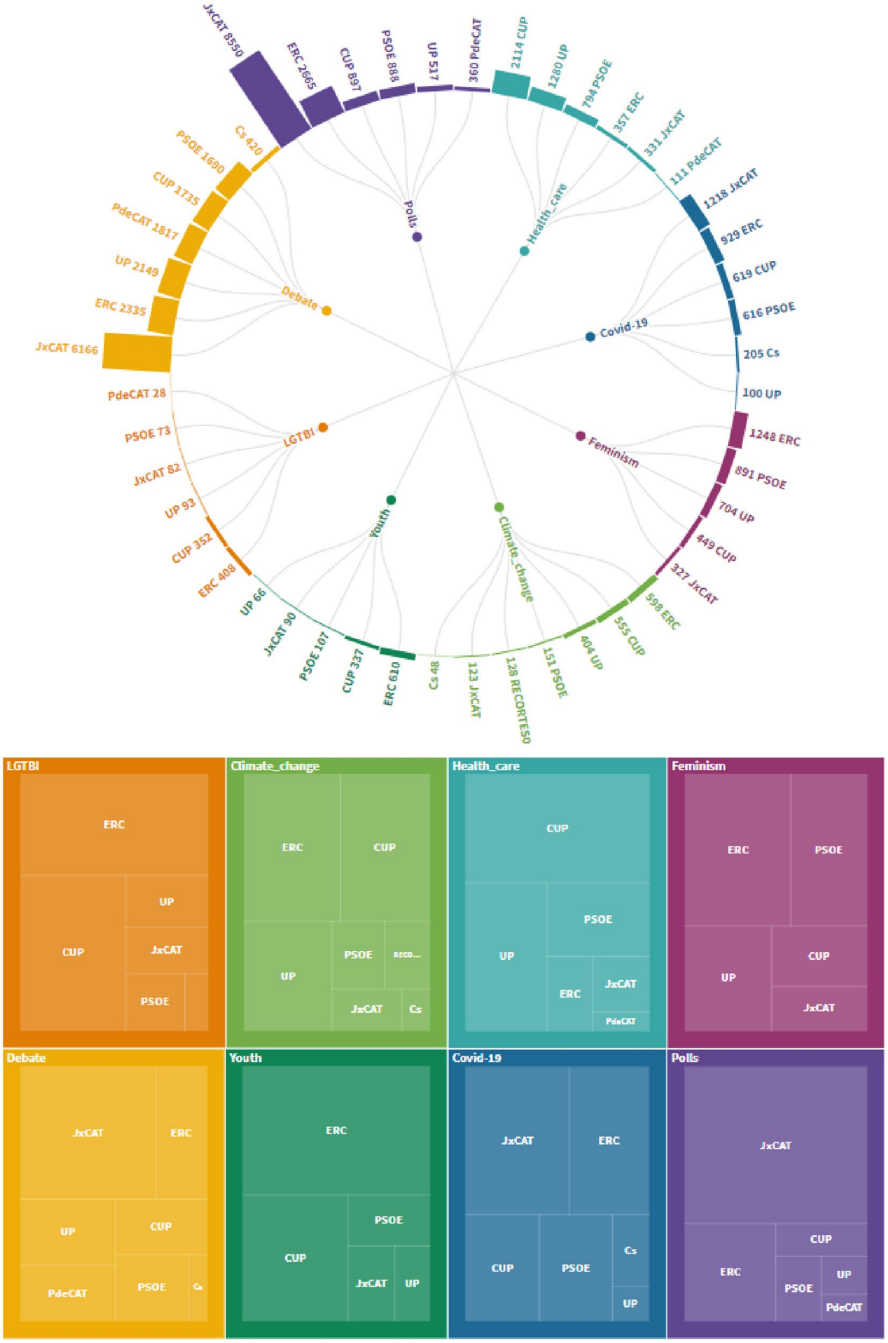
Presence of parties on key topics

When crossing the polarity with the topics, we can see that the communities are not exclusively echo-chambers, there are also interpellations allowing permeability in different communities (Fig. 7). In this sense, there are unifying topics among the left, whether they are independentists or not and whose polarity is positive. Perhaps it is for this reason that the lefts are closer in the graph. The environment, feminism, health, the LGTBI collective and youth are topics with the greatest presence among the left, even though in the global conversation of the elections JxCAT has many more tweets. Identifying the nodes (users) who spoke about the issues mentioned above, and painting them on the network (red colour), we see that the users of the communities on the left talk about the environment, feminism, LGTBI rights, public health and youth. Also, it is observed that the bridge between left-wing communities (nodes far from the centroids of the communities) retweets tweets of these topics.

On the right, there are very few topics in common between the Spanish national parties and the pro-independence ones. There is hardly any interpellation between communities. ‘VOX’ and ‘PP’, when they talk about independence, they have a negative speech. However, these tweets only represent 2% of the independence-related tweets. In the opposite case, ‘JxCat’ does enter the campaign hashtags of ‘VOX’ and ‘Cs’ with 5% and 46%, respectively. However, when commenting on the campaign tweets of the other two rights, he does so with a negative polarity, a 1.94 in VOX and a 2.07 in ‘Cs’.

## Discussion

### Revealing a multi-cleavage context by identifying topics discussed in social media

The influence of social media platforms on political and social polarization is a concern in many countries (Marozzo and Bessi 2018; Vicario et al. 2017). Academic realms like sociology, psychology, political science, or computational social science have extensively researched the social media echo-chambers (Everton 2016). One of the most prominent examples of the social media role in polarizing societies shows that Americans that deactivate their Facebook account are less polarized (Finkel et al. 2020). The main explanation for such result is that social media uses algorithms to filter and promote content that can maximize user engagement, which is usually content with high levels of evocation of emotions, reinforcing homophily in social networks and, therefore, enhancing political and social polarization.

Historically, the Spanish regions (e.g. Catalonia, Galicia and Basque Country) have been distinguished by cultural and linguistic diversity inside the Spanish nation. Each region has its own local language, which is also considered as official Spanish language. Therefore, Spain has 3 official languages which exist along with the Spanish nationwide, even if their origin is local.

But these additional official languages only spoken in specific region have participated to generate centrifugal forces from Madrid, capital and centre of Spain and to develop other regional political identities and new ideologies (e.g.: separatism). Moreover, the asymmetry in the socioeconomic situation between regions has favoured residents to claim for more autonomy and the cross-fertilization of a self-identification process and geographical echo-chambers (Dent 2017; Ruiz Vieytez 2019; Zaripova and Zakirov 2019).

In Catalonia, the grade of coexistence for both languages (Catalan and Spanish) has been instrumentalized is a marker of different ideologies and has developed a 3-tier cleavage context (federalism, unionism, and separatism). The acceptance of both languages as equivalent refers to federalism ideology, whereas unionists claim for the decrease of the Catalan importance in school and for its parity with Spanish in terms of teaching hours. On contrast, Separatists usually do not apply laws erected and voted by central government parliament, which could give more importance to Spanish in the public Catalan space (Khenkin 2018).

Although the topic has been extensively researched, most published investigations focus on the US context (Garimella et al. 2017). This generates a problem in the sense that since the political system in the USA is strongly partisan (i.e. two parties are in opposition), a generalizability of the results is quite difficult to do. For example, previous studies have concluded that social media activity in the USA has created a ‘mega-identity partisanship’ that differentiated online users not only by their political positions but also by their racial, religious, educational, or geographical characteristics (Primario et al. 2017).

Guth and Nelsen (2021) analyse that on top of a left–right cleavage an additional transnational cleavage exists between populists of right and left united against the traditional parties in their opposition to the ruling elites at home and in Brussels, and their approach in terms of security, identity, and community. However, the literature is scanty while considering other contexts where these characteristics are less airtight, and where we may find actors converging in some topics while diverging for others (Lee et al. 2018; Holmes and McNeal 2016). In this paper, to the best of our knowledge, we provide first evidence of this phenomenon. Indeed, we model in the context of a Spanish election a social media echo-chambers system in a multi-cleavage scenario.

So, our paper reveals that, in a situation of multiple political parties, echo-chambers are not always siloed as in the case of two-party conditions (Levendusky 2013). We argue it by analysing social media interactions in a multidimensional space beyond the obvious left–right dichotomy. Our findings highlight how social media behaviours differ depending on the cleavage put in focus. In other words, we show that echo-chambers and social media polarization are topic dependent (Nechushtai and Lewis 2019).

### Understanding the topics of cross-fertilization between social media echo-chambers

**Fig. 8:**
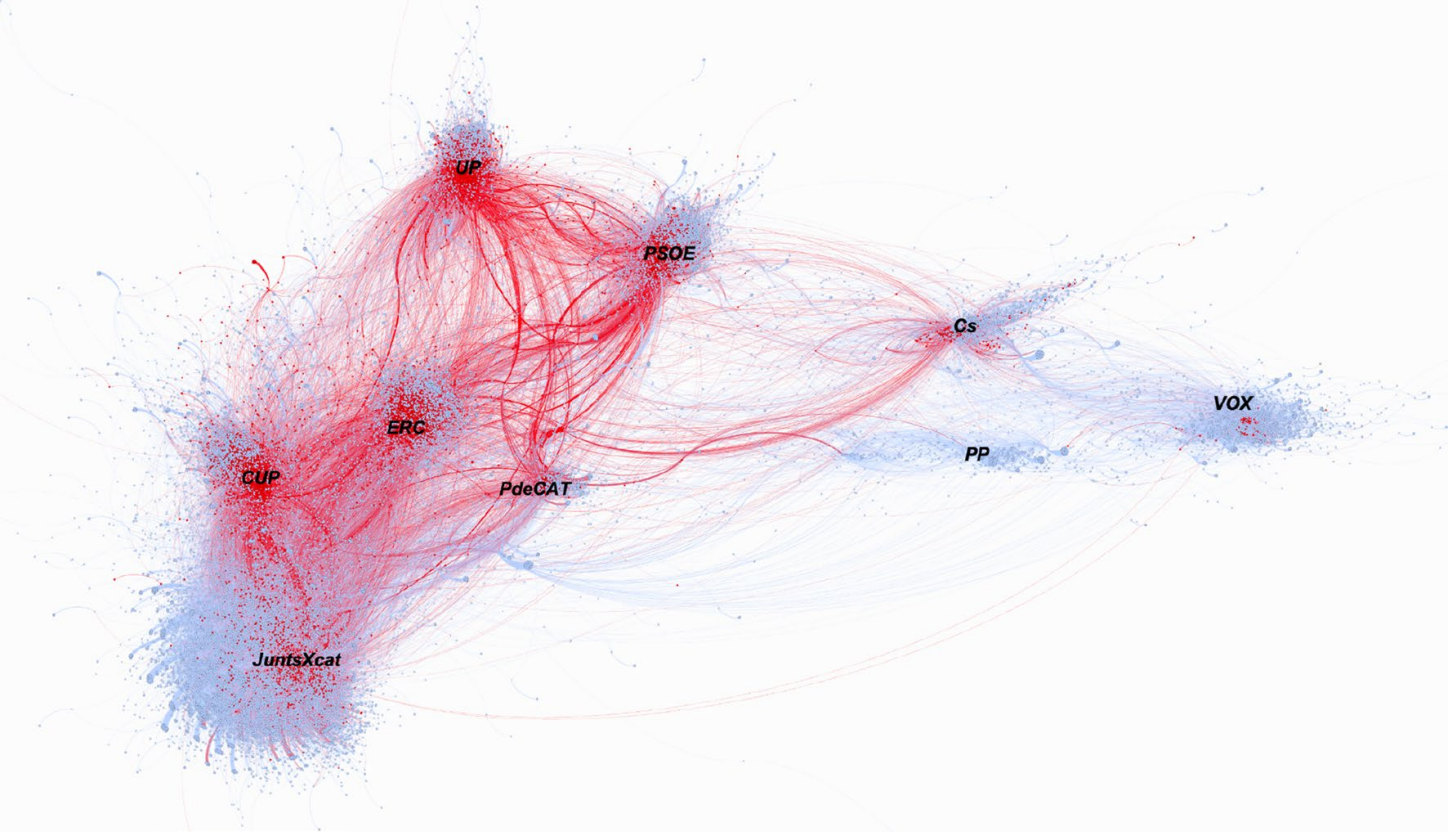
Graph of RTs on the Catalan campaign February 2021 coloured by left topic key (red colour) (colour figure online)

Our analysis demonstrates how social media contributes to increase polarization only in specific ways enabled by concrete themes. For instance, if some social media topics amplifies and escalates social divides (Buttliere and Buder 2017; Conover et al. 2011), we show that some others make converge social media users (Stromer-Galley 2003). In our context, we reveal that left-wing Spanish parties agree with left-wing Catalan parties on topics such as LGTBI rights or environmental policies while differing in the question of decentralization or independence right. More interestingly, Fig. 8 shows how right-wing independentist parties also agree and participate in conversations with the left-wing communities (both Spanish and Catalan) in topics like LGTBI rights, climate change or feminism. This contrasts with previous literature based in the USA where the dichotomy between left–right postulates is more clear-cut (Bail et al. 2018).

In addition, by adding a continuous scale of political positions (contrary to two-party settings), we show that far-right parties (‘*VOX*’) and Spanish right-wing parties (‘*PP*’* and *‘*Ciudadanos*’) do not participate in cross-group dialogues, while both Spanish and Catalan left-wing parties and Catalan right-wing parties do. Therefore, cross-fertilization and echo-chamber permeability is also a matter of choice and not only due to platform designs that nudge users towards content that reinforces previous positions (Ahlers 2006; Dutton et al. 2017; Newman et al. 2017). This has important implications for policy makers and social media companies alike.

There is extant discussion about the role of social media companies in fostering political polarization and how they should be regulated to avoid incentivizing extreme positions (Anderson and Huntington 2017; Demszky et al. 2019). However,in the light of our results we argue that the problem is not only with the design of these social media platforms (including their recommendation systems to enhance visibility of specific content), but also about how political parties use these platforms. While some actors may use the platform to engage in a democratic and fruitful dialogue, other actors may use the same platform either for magnifying their echo-chambers and political polarization to their advantage or propagating messages of incivility and sarcasm (Barberá 2014; Buder et al. 2021; Nithyanand et al. 2017).

We highlight the mechanisms of cross-fertilization in social media echo-chambers by showing combinations of interpellations merging polarity and topics, which allow permeability through different communities. Topics like LGTBI rights, climate change, health care (Jiang et al. 2021) or feminism are among the less polarizing position among parties. In these topics, we find positive conversations between right (only Catalan) and left-wing parties or between Catalan and Spanish parties. Indeed, for these issues, most Catalan nationalist parties agree along with half the Spanish nationalist parties. These results have important implications for social media platforms. First, they provide evidence that previous studies may overestimate the polarization effect of these platforms in countries outside the USA. Second, they show that the effects observed in the USA may be due, in part, to the specific political system (two-party) instead of only being a cause of social media platform designs and algorithms. Thirdly, these results point to the fact that different contexts require different legislations adapted to the specific context (Cadwalladr 2018). Our manuscript shows therefore the necessity of the creation of an online discursive space which legally protects citizens to manipulation (Iandoli et al. 2021).

### Identifying the polarity of sentiments in online out-group and in-group communication

The analysis of sentiment conveyed in the tweets (Fig. 5) shows a polarity difference between VOX (negative) to the rest of the parties (positive). Our results show the message of VOX is more oriented to the hate and negative feeling. Santamarina (2021) performs a semantic analysis of their discourse which reveals three types of messages: ultranationalism, racism and anti-feminism.

Through their negative behaviour in SM, VOX reanimates a climate of excluding nationalism. Similar to the language used during the Spanish Civil War and the subsequent dictatorship, they claim to be the ‘national force’ which fights with, what they call, the ‘anti-Spain’, the historical enemy of the Spanish nation: ‘los rojos’ (the reds) and the national minorities. These latter are now represented by a heterogeneous amalgam of movements that are jeopardizing the unity of the nation and the purity of the Spanish Catholic family (the feminists, the migrants, the Catalans, or the supporters of the left-wing party ‘UP’).

We observe that VOX develops this negative bias inside its community as a self-presentation to its own image (Milkman and Berger 2014). Our results cover the second research objective and show these negative sentiments seem to stay in VOX in-group communication and to only fertilize in this echo-chamber. In contrast, the rest of the parties share a positive bias in their form of communication.

Furthermore, beyond participating in conversations with the out-group, we observe that conversations are mainly positive for many actors. For example, Spanish left-wing parties and Catalan right-wing parties participate in conversations with Catalan left-wing parties in positive terms. This reinforces the ideas discussed in the previous paragraph: it is not that social media platforms are designed to increase polarity, but more as a matter of actor’s choice about how to use them (Kim et al. 2013). This may serve as an indication that political parties’ elites are more responsible for polarization than social networks (Levendusky 2017).

In general, emotions play a central role in the diffusion of online information, especially through social media. For example, Bail (2016) shows that posts containing highly emotional features were more viral, regardless the sentiment was positive or negative. Similarly, Brady et al. (2017) found that more emotional and more moral language increases the virality of tweets. This is especially important in the context outside the USA.

While the USA is a rigid two-party democratic system, most Western democracies are not. Of course, the more parties are there, the easier it is to contribute to out-group conversations. Our results show that there are systemic conditions that hinder polarization beyond social media, a finding that can be useful for the US context and other two-party political ecosystems.

## Conclusion

Our paper analyses the effect of echo-chambers in a multiparty context using Twitter data. It is one of the first empirical evidence to shed light into the ongoing discussion of social media polarization. While we do have evidence about social media effectively polarizing, we also have contrasting evidence showing that social media is fostering national conversations among opposing views. This manuscript presents evidence that the discussion may settle somewhere in between both the literature. In other words, the problem is not only social media per se, but how different actors use social media platforms.

Furthermore, geographical regions seeking independence is a highly topical theme present in Europe (e.g. Belgium, Italy, Spain, France, and UK), Asia (e.g. India or China) and also North America (e.g. Canada) (Taubman 1997; Mookherjee 2009; Baranov et al. 2016; Islami and Shamsabadi 2018; Okhoshin 2019). Lipovská (2020) has recently conducted a large survey across 17 European countries and 56 European regions. He analyses many secessionist movements; few of them have led to referendums such as in Catalonia, Scotland, Ukraine, or Kosovo. He concludes that sensibility of romantic factors such as language, religion or ethnicity are paramount in this process, along with rational and fact-based ones such as economic and political autonomy. His conclusions justify that the Spanish context is representative of a diverse nation and therefore our results could be extended beyond Spain into similar contexts.

Of course, our study has the limitation of being focused on a specific context and may not be generalizable to other settings. However, it is one of the first to show that, outside the U.S. context (where most of the previous literature focuses) there is space for more research refinement. This opens the door for future research about more contexts and other topics, because at the end, both context and topics are the drivers of political polarization and echo-chambers, not just social media platform design.

We believe that further research should analyse if our results are applicable to other social media platforms like Instagram or Facebook. Moreover, we collected data during one of the most polarizing moments of any democratic society: the electoral campaign. This may be also a limitation when analysing social media data in a different period, not such “easy” to the observation of the society. Even considering the aforementioned limitations, we do believe that our manuscript shows polarization still exist even if the size of this polarization is not as big as in other contexts, mainly the over-studied US context. Considering these conditions, we corroborate, however, the necessity of the creation of a higher quality online discursive space, able to resist manipulation (Iandoli et al. 2021).

## Appendix 1: Monitored hashtags

#14F

#14FComenÁaElCanvi

#alcostatdelagent

#alvostrecostat

#CampaÒaCataluÒa

#CentremElPaÌs

#CienciaCatMereix

#DBTLaVanguardia

#DebateCATrtve

#ElCanviQueCatalunyaMereix

#Femho

#FemHoJunts

#GanaIlla

#Hag·moslo

#Hi…remHiSomHiSerem

#Junts

#JuntsPerFer

#JuntsPerGuanyar

#JuntsPerSer

#noucicle

#nouclicle

#ParaQueGanemosTodos

#PerGuanyar

#polÌtica˙til

#PorUnaEducaciÛnLibre

#PrenemElRelleu

#PresidentIlla

#RecuperemosCataluÒa

#Rep˙blicaCatalana

#SiThoPensesPDeCAT

#StopIslamizaciÛn

#TornaCatalunya

#UnaCatalunyaMillor

#unacataluÒamejor

#UnaCataluÒaParaTODOS

#UnNouCicle

#viaamplia

#VotaVox

#VotoPorCorreo14F

#AquÌDebat

#VoxExtremaNecesidad

#StopDespilfarro

#VoxLivesMatter

#BarriosSeguros

#DebatTV3

#L6ElDebat

#14FTV3

#Elecciones14F

#EleccionesCatalanas

#Eleccions14F

#RompeTuVot

#YoNoVoto14F

#StopIslamizacion

## Appendix 2: Guideline table of party ideologies

In order to contextualize the ideological position (left vs right and independence vs centralism) of the different parties that appear in the analysis, a descriptive table has been added below. The low scale (value of 1) is associated with parties with a left-wing ideology, as well as supporters of Catalonia independence. On contrary, the high scale (value of 10) is related to parties with right-wing ideology and supporters of Spanish national centralism.

**Table:**

Party	Left (1) vs Right (10)	Independence (1) vs Centralism (10)
VOX	9.4	10
PP	7.9	9.7
Cs	6.9	9.5
PSOE	3.8	5.6
ERC	3.1	1.3
UP	2.3	3.5
PdeCAT	6.5	4.2
JxCAT	6	2
CUP	1.5	1

The table has been developed with data from the Spanish Statistical Office (CIS 2020) and (Colomer 2017: Global Security 2021).

## Declarations

### Data availability

The dataset collected and analysed during the current study is available from the corresponding author on reasonable request.
